# Characteristics and Outcomes in Patients with Ventilator-Associated Pneumonia Who Do or Do Not Develop Acute Respiratory Distress Syndrome. An Observational Study

**DOI:** 10.3390/jcm9113508

**Published:** 2020-10-29

**Authors:** Enric Barbeta, Adrian Ceccato, Antoni Artigas, Miquel Ferrer, Laia Fernández, Rubén López, Leticia Bueno, Anna Motos, Gianluigi Li Bassi, Ricard Mellado, Carlos Ferrando, Andrea Catalina Palomeque, Mauro Panigada, Albert Gabarrús, Diego de Mendoza, Antoni Torres

**Affiliations:** 1 Department of Pneumology and Respiratory Intensive Care Unit, Institut Clinic de Respiratori, Hospital Clinic of Barcelona, 08036 Barcelona, Spain; barbeta@clinic.cat (E.B.); miferrer@clinic.cat (M.F.); lfernan1@clinic.cat (L.F.); rlopeza@clinic.cat (R.L.); bueno@clinic.cat (L.B.); amotos@clinic.cat (A.M.); palomeque@clinic.cat (A.C.P.); gabarrus@clinic.cat (A.G.); 2 Institut d’Investigacions Biomèdiques August Pi i Sunyer (IDIBAPS), University of Barcelona (UB), Ciber de Enfermedades Respiratorias (Ciberes, CB06/06/0028), 08036 Barcelona, Spain; aceccato@clinic.cat; 3 Intensive Care Unit, Hospital Universitari Sagrat Cor, CIBER Enfermedades Respiratorias, 08036 Barcelona, Spain; diego.mendoza@quironsalud.es; 4 Critical Care Center, Corporación Sanitaria Universitaria Parc Tauli, CIBER Enfermedades Respiratorias, Autonomous University of Barcelona, 08208 Sabadell, Spain; aartigas@tauli.cat; 5 Critical Care Research Group, The Prince Charles Hospital, Brisbane, QLD 4032, Australia; glibassi@clinic.cat; 6 Department of Anesthesiology and Surgical Intensive Care Unit, Hospital Clinic of Barcelona, 08036 Barcelona, Spain; rmellado@clinic.cat (R.M.); cmferrando@clinic.cat (C.F.); 7 Anesthesia, Intensive Care and Emergency Department, Foundation IRCCS Ca’ Granda Ospedale Maggiore Policlinico, 20122 Milano, Italy; mauropanigada@gmail.com

**Keywords:** ventilator-associated pneumonia, acute respiratory distress syndrome, epidemiology

## Abstract

Ventilator-associated pneumonia (VAP) is a well-known complication of patients on invasive mechanical ventilation. The main cause of acute respiratory distress syndrome (ARDS) is pneumonia. ARDS can occur in patients with community-acquired or nosocomial pneumonia. Data regarding ARDS incidence, related pathogens, and specific outcomes in patients with VAP is limited. This is a cohort study in which patients with VAP were evaluated in an 800-bed tertiary teaching hospital between 2004 and 2016. Clinical outcomes, microbiological and epidemiological data were assessed among those who developed ARDS and those who did not. Forty-one (13.6%) out of 301 VAP patients developed ARDS. Patients who developed ARDS were younger and presented with higher prevalence of chronic liver disease. Pseudomonas aeruginosa was the most frequently isolated pathogen, but without any difference between groups. Appropriate empirical antibiotic treatment was prescribed to ARDS patients as frequently as to those without ARDS. Ninety-day mortality did not significantly vary among patients with or without ARDS. Additionally, patients with ARDS did not have significantly higher intensive care unit (ICU) and 28-day mortality, ICU, and hospital length of stay, ventilation-free days, and duration of mechanical ventilation. In summary, ARDS deriving from VAP occurs in 13.6% of patients. Although significant differences in clinical outcomes were not observed between both groups, further studies with a higher number of patients are needed due to the possibility of the study being underpowered.

## 1. Introduction

Ventilator-associated pneumonia (VAP) is a complication that arises in patients on invasive mechanical ventilation. Its incidence is 9.3% among those patients mechanically ventilated longer than 24 hours; however, it can reach as high as 50% in trauma and brain injury patients [1,2]. The incidence density is 9.83 per 1000 ventilator days but can be decreased with preventive measures [3]. The most common causative pathogens in Europe and the United States are *Pseudomonas aeruginosa* and *Staphylococcus aureus* [4]. VAP is associated with poor clinical and economic outcomes when compared to mechanically-ventilated controls [2]. These patients present with longer duration of mechanical ventilation, further intensive care unit (ICU) and hospital length of stay, and increased costs. VAP accounts for an attributable mortality of 13%, driven mainly by surgical patients and those with an intermediate severity of illness score [5]. 

Acute respiratory distress syndrome (ARDS) manifests as non-cardiogenic bilateral pulmonary opacities that cause respiratory failure with a PaO_2_/FiO_2_ ≤ 300 in patients undergoing mechanical ventilation [6]. ARDS is a potential complication deriving from VAP, as it may induce bilateral pulmonary infiltrates and severe respiratory failure, which fulfills the Berlin criteria for ARDS diagnosis [6]. ARDS mortality can reach as high as 45% in the most severe cases despite advances in ventilator management and supportive care [7]. 

VAP and ARDS share pathophysiological mechanisms that can contribute to lung injury. Both pathogen-associated virulence factors found in VAP and ventilation-induced lung injury associated with ARDS cause pulmonary inflammation [8,9,10]. Excessive inflammation hampers the neutralization and clearance of bacteria in the lungs and enhances bacterial growth [11]. 

Indeed, VAP is a well-known and frequent complication in patients with ARDS [12]; however, data regarding the incidence of ARDS as the initial manifestation of VAP, as well as related pathogens and specific outcomes is limited. Our hypothesis is that patients with VAP and ARDS might have different clinical features and outcomes when compared to patients with VAP who do not have ARDS. Knowing incidence and possibly different clinical characteristics, microbiology, or outcomes of patients with VAP and ARDS could result in benefits due to specific clinical management. Thus, we aimed to assess and compare incidence, microbiology, and clinical outcomes among patients with VAP who developed ARDS in comparison to those who did not. 

## 2. Methods

### 2.1. Design and Patients

This cohort study evaluated patients with VAP in six ICUs of an 800-bed tertiary teaching hospital (Hospital Clinic, Barcelona, Spain), who were admitted between 2004 and 2016. Investigators conducted daily rounds in the ICUs. Patients were included in the study if they were ≥18 years and presented with clinically suspected pneumonia 48 hours after ICU admission. Patients with severe immunosuppression were excluded, including those underdoing solid organ or hematopoietic transplantation or chemotherapy; those with drug-induced immunosuppression; or those with human immunodeficiency virus infection. Only the first episode of pneumonia of each patient was assessed. 

The study was approved by the Institution’s Internal Review Board for publishing (approval number 5427) and written informed consent was obtained from either patients or their relatives. All data were collected according to a standardized clinical protocol and retrospectively analyzed.

### 2.2. Definition of Pneumonia and ARDS

**Figure 1:**
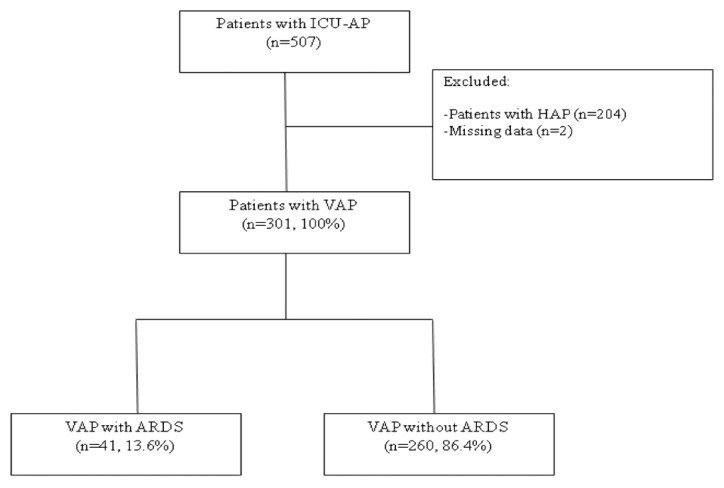
Flow diagram of the study population. Abbreviations: ARDS, acute respiratory distress syndrome; HAP, hospital-acquired pneumonia; ICU-AP, intensive care unit-acquired pneumonia; VAP, ventilator-associated pneumonia.

Pneumonia was clinically diagnosed in patients who presented with new or progressive pulmonary infiltrates in their chest radiographs due to a presumed infectious agent, and at least two of the following symptoms or findings: fever (>38 °C) or hypothermia (<36 °C), leukocytosis (>12,000 cells/mm^3^) or leukopenia (<4000 cells/mm^3^), presence of purulent tracheal secretions, and a decrease in oxygenation [2]. VAP was defined in patients who were treated with invasive mechanical ventilation for ≥ 48 hours and had met the criteria. Patients presenting with hospital-acquired pneumonia without invasive mechanical ventilation were excluded from the study (Figure 1). 

ARDS was defined as bilateral opacities in x-ray or computed tomography (CT) not fully explained by effusions, lung/lobar collapse or nodules; the ruling out of cardiac failure or fluid overload; and PaO_2_/FIO_2_ ≤ 300 mmHg with positive-end expiratory pressure (PEEP) or continuous positive airway pressure (CPAP) ≥ 5 cmH_2_O. The severity of ARDS was divided into three categories per the Berlin Definition [6]: mild (200 <PaO_2_/FIO_2_ ≤ 300 mmHg), moderate (100 < PaO_2_/FIO_2_ ≤ 200 mmHg), and severe (PaO_2_/FIO_2_ ≤ 100 mmHg). Patients diagnosed with the previous definition were assessed again, according to updated Berlin Definition.

### 2.3. Microbiological Evaluation and Diagnosis Criteria

We collected at least one respiratory sample of tracheobronchial aspirates, protected specimen brush, or bronchoalveolar lavage when VAP was suspected. Respiratory samples were processed for Gram and Ziehl–Neelsen staining, as well as for bacterial, fungal, and mycobacterial cultures and viral identification analyses. Criteria for etiological diagnosis were described by pre-defined thresholds for quantitative cultures (i.e., protected specimen brush >10^3^, bronchoalveolar lavage >10^4^, and tracheobronchial aspirates >10^5^ colony-forming unit/mL, respectively) and the presence of microorganisms for qualitative cultures [13].

### 2.4. Data Collection

All relevant data were collected at ICU admission and upon onset of pneumonia, as noted in medical records and bedside flow charts i.e., clinical, laboratory, radiological, and microbiological information. Patient follow-up was extended to death, hospital discharge, or up to 90 days after diagnosis of pneumonia. Severity was assessed during ICU admission and at VAP diagnosis, using both the Sequential Organ Failure Assessment (SOFA) score [14] and Simplified Acute Physiology Score (SAPS) II [15]. 

### 2.5. Antimicrobial Treatment

Initial empirical antibiotic therapy was administered according to the attending physician’s recommendations. Local policy and practice were based mainly on an adaptation of the American Thoracic Society (ATS)/Infectious Diseases Society of America (IDSA) guidelines [16]. It was also recommended that physicians based their empirical choices on the local prevalence of multidrug-resistant (MDR) pathogens. Local policy also recommended re-evaluating antibiotic regimens based upon culture results. Empirical antibiotic treatment was defined as appropriate when cultured pathogens tested sensitive in antibiotic sensitivity analyses. 

### 2.6. Outcome Measures

The primary outcome measurement was mortality on day 90 after the onset of VAP; secondary outcome measurements were 28-day mortality, ICU mortality, ventilation-free days, ICU and hospital length of stay, and duration of mechanical ventilation. To assess the influence of timespans on primary and secondary outcomes, a sub-analysis was performed, dividing the 12-year cohort into four time spans. 

### 2.7. Statistical Analysis

We reported the number and percentage of patients for categorical variables, median and first and third quartile (Q1; Q3) for continuous variables with non-normal distribution and mean and standard deviation for those with a normal distribution. Categorical variables were compared using the χ^2^ test or Fisher exact test. Continuous variables were compared using the t-test or nonparametric Mann–Whitney U test.

Survival curves of patients with and without ARDS were obtained using the Kaplan–Meier method and compared using the Gehan–Breslow–Wilcoxon test. Cox proportional-hazards regression analyses were used to evaluate the effect of several factors on survival simultaneously [17]. In the first phase, each risk factor was tested individually. In the second phase, all risk factors that showed an association in the univariate model (p < 0.10) were added to the multivariable model. Finally, a backward stepwise selection (p_in_ < 0.05, p_out_ > 0.10) was used to determine factors associated with mortality. Multicollinearity was confirmed by calculating the variance inflation factor. We calculated hazard ratios (HRs) and their 95% confidence intervals (CIs). Proportional hazard assumptions were tested with log-minus-log plots. To evaluate the lack of fit of our final model, we evaluated deviance residuals.

To measure possible overfitting and the instability of selection variables in our final models, we performed internal validation using ordinary nonparametric bootstrapping with 1000 bootstrap samples and bias-corrected and accelerated 95% CIs [18].

The multiple imputation method [19] was used to handle missing data in the regression analyses.

The level of significance was set at 0.05 (two-tailed).

All analyses were performed using IBM SPSS Statistics version 25.0 (Armonk, New York, NY, USA).

## 3. Results

### 3.1. Patient Characteristics

Out of the 301 mechanically ventilated patients with VAP, 41 (13.6%) developed ARDS (Table 1 and Figure 1). The median (Q1; Q3) time from endotracheal intubation (ETI) to VAP diagnosis was 5 (3; 9) days for ARDS patients and 6 (3; 10) days for non-ARDS patients (p = 0.79). On average, those patients who developed ARDS were younger and presented with lower SAPS-II scores upon ICU admission (p = 0.025) but higher SOFA at VAP diagnosis (p = 0.001). In addition, patients with VAP and ARDS had chronic liver disease more frequently (p = 0.014) but less chronic obstructive pulmonary disease (COPD) (p = 0.033). Main causes of ICU admission did not differ between VAP patients with and without ARDS (Table 1).

With regards to severity, moderate ARDS (100 < PaO_2_/FIO_2_ ≤ 200 mmHg) occurred the most frequently (n = 29, 70.7%); six patients (14.6%) presented with mild ARDS (200 < PaO_2_/FIO_2_ ≤ 300 mmHg); and the remaining six patients (14.6%) presented with severe ARDS (PaO_2_/FiO_2_ ≤ 100 mmHg). Excluding a mild increase in the median respiratory rate in patients with ARDS, mechanical ventilation parameters (i.e., PEEP, tidal volume, etc.) did not differ between the two groups. Respiratory mechanics analysis did not show any difference among these patients.

### 3.2. Microbiological Diagnosis

A microbial diagnosis was obtained from 215 patients (71.4%) (Table 2). The most frequent pathogen was *Pseudomonas aeruginosa* without any difference in both groups (p = 0.16), including MDR *Pseudomonas aeruginosa* (p = 0.089). Appropriate empirical antibiotic treatment was prescribed for ARDS patients as frequently as for those who did not present with ARDS (76% and 85.8%, respectively; p = 0.20). 

### 3.3. Mortality, Length of Stay, and Ventilator-Free Days 

**Figure 2:**
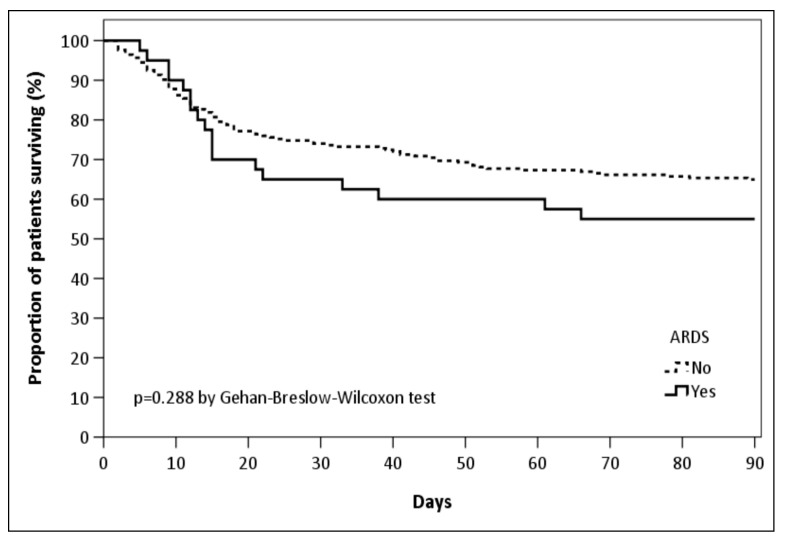
Kaplan–Meier survival curves at 90 days.

We did not find any differences in ICU, 28- and 90-day mortality between patients who did or did not develop ARDS. Ventilator-free days, and ICU and hospital length of stay also did not differ between the groups (Table 3 and Figure 2).

ARDS was not associated with 28- and 90-day mortality in multivariate analyses (Supplemental Tables S1 and S2). Active neoplasm, higher SOFA score, and shock at the onset of VAP, chronic heart disease, chronic liver disease, age and corticosteroid treatment before VAP were independent factors associated with 90-day mortality. Internal validation by bootstrapping with 1000 samples confirmed robust results for the variables included in the multivariable models, with small 95% CIs around the original coefficients. We analyzed these outcomes in four time spans and did not find any differences (Supplementary Materials Tables S3–S6).

## 4. Discussion

The main findings of this study are the following: ARDS was present in 13.6% of patients with VAP in a single-center, consecutively collected cohort. The presence of ARDS did not cause significantly higher ICU, 28- and 90-day mortality, ICU, and hospital length of stay, or less ventilation-free days. The most frequent causative pathogen was *Pseudomonas aeruginosa*, without any differences between both VAP groups. Patients with ARDS were found to be more severely injured at VAP diagnosis, accounting for a higher SOFA score. 

To our knowledge, this is the largest cohort study of patients with VAP presenting with ARDS. ARDS is a well-known clinical problem in the ICU, frequently resulting from a respiratory infection and presenting with both high prevalence (10.4%) and mortality (34.9–46.1%) [7]. Although it has been studied in community-acquired pneumonia (CAP), data regarding its impact on VAP remains scarce [20]. Similar to CAP patients admitted to the ICU, the incidence of ARDS was 13.6%, and no association was found with mortality [20]. Only one study has analyzed the impact of ARDS on VAP. It reported that ARDS delays clinical resolution (fever, radiographic opacities, white blood cell count, and hypoxemia) without clearly affecting mortality [21]. Clinical resolution delay may hamper liberation from mechanical ventilation in this subset of patients. However, in our study, as it concerned regarding less ventilation-free days or ICU and hospital length of stay, we did not find significant differences between VAP patients with or without ARDS.

Other studies have analyzed ARDS within a hospital setting. Hospital-acquired ARDS was studied in this large study by Ahmed et al [22]; nosocomial pneumonia accounted for 20–25% of cases. Unlike our study and those mentioned prior [21], ARDS was associated with a higher risk of mortality. 

Previous research found a greater proportion of *Pseudomonas aeruginosa* in ARDS deriving from VAP when compared to controls [21]. In our study, microbiological etiology was not different between both groups of VAP, including *Pseudomonas aeruginosa* and its MDR strains. Further, no differences were found in antibiotic empirical treatment adequacy per etiological diagnosis. 

Liver cirrhosis is a known risk factor for ARDS development in critically ill patients [23]. In our cohort, chronic liver disease was also found to be more frequent in patients with VAP and ARDS. However, COPD was not found in VAP patients with ARDS as frequently as in those without ARDS. This finding was unexpected and difficult to interpret. We found a significantly higher proportion (19% vs. 7%) of patients with prior ARDS diagnosis who later developed VAP and fulfilled the criteria for ARDS diagnosis upon VAP diagnosis. In these patients, VAP occurred in lungs with a preexistent bilateral injury, which made meeting the Berlin criteria more likely. It is difficult to establish to what extent this new ARDS is caused by VAP; this underpins the limitations inherent in VAP and ARDS diagnosis.

Mechanical ventilation parameters were not different between both groups, except for a mild increase in the respiratory rate in the ARDS group (which may not be clinically relevant). On average, the most relevant variables related to ventilation-induced lung injury, that is, driving pressure and plateau pressure, were within normal limits in both groups. 

The limitations of this study are the following. First, this study was done in a single-center cohort and the results need to be validated. Second, despite a large set of VAP patients, we could only analyze 41 patients with VAP-ARDS. This sample size may result in a large type II error and conclusions that can be drawn are limited. Third, we only analyzed the first case of ARDS after VAP diagnosis. Fourth, despite the inclusion of a subgroup analysis by timespan, we acknowledge that several changes have been implemented in the management and prevention of VAP and ARDS throughout the study duration [24,25,26,27]. Accordingly, the generalizability of the main results may be limited. These limitations notwithstanding, the rigorous approach to the study underpins our confidence in its findings and their clinical relevance.

## 5. Conclusions

ARDS deriving from VAP occurs approximately in 14% of patients. In this single-center study, we did not find significant differences in mortality, ICU, and hospital length of stay, and ventilation-free days in patients with or without ARDS. However, due to limited sample size, this study might be underpowered in detecting these differences and further studies with a higher number of patients are thereby warranted. 

## List of Abbreviations

ARDSAcute respiratory distress syndromeATSAmerican thoracic societyCAPCommunity acquired pneumoniaCIConfidence intervalCOPDChronic obstructive pulmonary diseaseCPAPContinuous positive airway pressure CTComputed tomographyETIEndotracheal intubation HRHazard ratios ICUIntensive care unitIDSAInfectious Diseases Society of AmericaMDRMultidrug-resistant pathogensPEEPPositive end-expiratory pressureQ1First quartileQ3Third quartileSAPSSimplified acute physiology score SOFASequential organ failure assessmentVAPVentilator associated pneumonia

## Figures and Tables

**Table 1:** Baseline characteristics, main causes of intensive care unit admission; severity and respiratory mechanics of the patients.

Variables	Patients with Ventilator-Associated Pneumonia Who Developed Acute Respiratory Distress Syndrome *n* = 41	Patients with Ventilator-Associated Pneumonia Who Did not Develop Acute Respiratory Distress Syndrome *n* = 260	p-Value
Age, years, median (Q1; Q3)	56 (47; 69)	66 (53; 74)	0.020
Sex, male/female, *n*	23/18	184/76	0.060
Alcohol abuse (current or former), *n* (%)	8 (20)	63 (24.2)	0.55
Prior corticosteroid use, *n* (%)	4 (10)	25 (10.6)	>0.99
SAPS II score at ICU admission, median (Q1; Q3)	35.5 (27; 44)	42 (32; 53)	0.025
SOFA score at ICU admission, median (Q1; Q3)	7 (6; 9)	8(5; 10)	0.95
**Comorbidities, *n* (%) ^a^**			
Diabetes Mellitus	7 (17.1)	63 (24.2)	0.31
Chronic renal failure	2 (4.9)	23 (8.8)	0.54
Chronic heart disease	8 (19.5)	90 (34.6)	0.055
Chronic lung disease	11 (26.8)	81 (31.2)	0.57
COPD	3 (7.3)	56 (21.5)	0.033
Solid neoplasm	6 (14.6)	28 (10.8)	0.43
Chronic liver disease	11 (26.8)	32 (12.3)	0.014
**Main causes of ICU admission and Severity, *n* (%)**			
Post-operative	7 (17.1)	55 (21.2)	0.54
Decreased consciousness	5 (12.2)	45 (17.3)	0.41
Hypoxemic respiratory failure	5 (12.2)	23 (8.8)	0.56
Multiple traumas	3 (7.3)	25 (9.6)	0.77
Hypercapnic respiratory failure	7 (17.1)	23 (8.8)	0.15
Septic shock	4 (9.8)	21 (8.1)	0.76
Cardiogenic shock	-	4 (1.5)	0.23
Hypovolemic shock	3 (7.3)	3 (1.2)	0.23
Abdominal disorders	2 (4.9)	9 (3.5)	0.65
Cardiac arrest	2 (4.9)	24 (9.2)	0.55
Acute coronary syndrome	-	14 (5.4)	0.23
Other	3 (7.3)	14 (5.4)	0.23
Days from ETI to VAP, median (Q1; Q3)	5 (3; 9)	6 (3; 10)	0.79
SOFA at VAP onset, median (Q1; Q3)	9 (8; 11)	7 (5; 10)	0.001
Shock at VAP onset, *n* (%)	24 (58.5)	123 (47.7)	0.20
PaO_2_/FiO_2_, median (Q1; Q3)	145 (126.2; 182)	212 (152; 272.5)	<0.001
Prior ARDS diagnosis, *n* (%)	8 (19)	17 (7)	0.005
**Mechanical ventilation parameters and respiratory mechanics, median (Q1; Q3)**			
Tidal volume (mL)	490 (428; 527)	500 (450; 565)	0.18
Respiratory rate (respirations per minute)	18 (15; 20)	16 (14; 19)	0.012
Positive end-expiratory pressure (cmH_2_O)	6 (5; 10)	7.5 (5; 9)	0.90
End-inspiratory pressure (plateau pressure) (cmH_2_O)	20 (16; 26)	21 (17; 25)	0.96
Respiratory system compliance (mL×cmH_2_O^−1^)	38.5 (30.7; 68.5)	40 (29.6; 54)	0.91
Driving pressure (cmH_2_O)	13 (8; 16)	14 (10; 17)	0.51

Patients’ characteristics; severity at ICU admission and comorbidities of 301 VAP patients with and without ARDS. Abbreviations: ARDS, acute respiratory distress syndrome; COPD, chronic obstructive pulmonary disease; ETI, endotracheal intubation ICU, intensive care unit; Q1, first quartile; Q3, third quartile; SAPS, simplified acute physiology score; SOFA, sequential organ failure assessment; VAP, ventilator-associated pneumonia. Percentages calculated with non-missing data only. ^a^ Possibly > 1 comorbidity.

**Table 2:** Pathogen etiology.

Microbiology, *n* (%)	Patients with Ventilator-Associated Pneumonia Who Developed Acute Respiratory Distress Syndrome *n* = 41	Patients with Ventilator-Associated Pneumonia Who Did not Develop Acute Respiratory Distress Syndrome *n* = 260	p-Value
Microbiological diagnosis	25 (61)	190 (73.1)	0.11
*Pseudomonas aeruginosa*	14 (34.1)	62 (23.8)	0.16
*Pseudomonas aeruginosa* MDR	7 (17.1)	17 (6.5)	0.089
Polymicrobial ^a^	7 (18.9)	47 (18.8)	0.99
*Staphylococcus aureus* MSSA	5 (13.5)	56 (22.4)	0.22
*Staphylococcus aureus* MRSA	2 (4.9)	16 (6.2)	0.58
*Klebsiella* spp	2 (5.4)	23 (9.2)	0.75
*Escherichia coli*	3 (8.1)	10 (4)	0.23
*Aspergillus* spp	2 (5.4)	5 (2)	0.22
*Proteus* spp	1 (2.7)	5 (2)	0.57
*Enterobacteriaceae* spp	1 (2.7)	12 (4.8)	>0.99
*Serratia* spp	1 (2.7)	9 (3.6)	>0.99
*Pneumoccocus* spp	1 (2.7)	9 (3.6)	>0.99
*Stenotrophomona maltophilia*	1 (2.7)	12 (4.8)	>0.99
Virus	2 (5.4)	3 (1.2)	0.13
Appropriate empirical treatment	19 (76)	163 (85.8)	0.20

Etiological diagnosis of pneumonia in 215 patients with positive microbiology. Appropriate empirical treatment. Abbreviations: ARDS, acute respiratory distress syndrome; MDR, multidrug-resistant; MSSA methicillin-sensitive Staphylococcus aureus; MRSA, methicillin-resistant Staphylococcus aureus; VAP, ventilator-associated pneumonia. Percentages calculated with non-missing data only. ^a^ Polymicrobial pneumonia was defined as those with more than 1 potential pathogen identified.

**Table 3:** Clinical outcomes.

Variables	Patients with Ventilator-Associated Pneumonia Who Developed Acute Respiratory Distress Syndrome *n* = 41	Patients with Ventilator-Associated Pneumonia Who Did not Develop Acute Respiratory Distress Syndrome *n* = 260	p-Value
Outcomes			
28-day mortality, *n *(%)	12 (29.3)	58 (22.3)	0.32
90-day mortality, *n* (%)	18 (45)	89 (35)	0.22
ICU mortality, *n* (%)	15 (36.6)	66 (25.4)	0.13
ICU length of stay (days), median (Q1; Q3)	21 (13; 41)	19 (13; 31)	0.26
Hospital length of stay (days), median (Q1; Q3)	37 (21; 63)	37.5 (21; 61.5)	0.59
28-day ventilator-free days, median (Q1; Q3)	0 (0; 17.5)	12 (0; 22)	0.10
Duration of mechanical ventilation (days), median (Q1; Q3)	18 (11; 33)	14 (9; 24)	0.11

Clinical outcomes of 301 VAP patients with and without ARDS. Abbreviations: ARDS, acute respiratory distress syndrome; ICU, intensive care unit; Q1, first quartile; Q3, third quartile; VAP, ventilator-associated pneumonia. Percentages calculated with non-missing data only.

## Supplementary Materials

The following are available online at https://www.mdpi.com/2077-0383/9/11/3508/s1, Table S1. Significant univariate and multivariate Cox regression analyses of variables associated with 28-day mortality, Table S2. Significant univariate and multivariate Cox regression analyses of variables associated with 90-day mortality, Table S3. Clinical outcomes of 72 patients with VAP who did and did not develop ARDS during 2004–2007, Table S4. Clinical outcomes of 113 patients with VAP who did and did not develop ARDS during 2008–2010, Table S5. Clinical outcomes of 81 patients with VAP who did and did not develop ARDS during 2011–2013, Table S6. Clinical outcomes of 35 patients with VAP who did and did not develop ARDS during 2014–2016.
