# Supplementary material for: Characteristics and Outcomes in Patients with Ventilator-Associated Pneumonia Who Do or Do Not Develop Acute Respiratory Distress Syndrome. An Observational Study
Source: J Clin Med. 2020 Oct 29;9(11):3508. doi: 10.3390/jcm9113508 (PMC7692126; doi:10.3390/jcm9113508)
Supplement: Supplementary file 1 [file jcm-09-03508-s001.pdf]

Journal of
Clinical Medicine

MDPI

Table S1. Significant univariate and multivariate Cox regression analyses of variables associated with 28-day mortality.

Variable	Univariate[a]			Multivariable		
	HR	95% CI	p-value	HR	95% CI	p-value
ARDS	1.27	0.68–2.37	0.45	1.03	0.55–1.95	0.92
Active neoplasm	1.96	1.08–3.59	0.028	-	-	-
Chronic heart disease	0.30	0.15–0.58	<0.001	0.30	0.15–0.60	0.001
Chronic lung disease	1.62	1.00–2.60	0.048	2.01	1.24–3.26	0.005
Chronic liver disease	2.09	1.21–3.62	0.008	1.78	1.02–3.09	0.043
SOFA at VAP onset	1.11	1.04–1.18	0.001	-	-	-
Shock at VAP onset	2.22	1.35–3.64	0.002	2.10	1.28–3.45	0.003

Significant univariate and multivariate Cox regression analyses of variables associated with 28-day mortality. Abbreviations: ARDS, acute respiratory distress syndrome; CI, confidence interval; HR, hazard ratio; SOFA, sequential organ failure assessment, VAP, ventilator-associated pneumonia. Data are shown as estimated HRs (95% CIs) of the explanatory variables in the 28-day mortality group. The HR is defined as the ratio of the hazard rates corresponding to the conditions described by two levels of an explanatory variable (the hazard rate is the risk of death [i.e., the probability of death], given that the patient has survived up to a specific time). The P-value is based on the null hypothesis that all HRs relating to an explanatory variable equal unity (no effect). [a] The variables analysed in the univariate analysis were age, sex, corticosteroid use before VAP, infection (respiratory and non-respiratory) before VAP, diabetes mellitus, chronic renal failure, chronic heart disease, chronic lung disease, active solid neoplasm, chronic liver disease, SOFA and shock at VAP onset, creatinine, hemoglobin, lymphocytes and C-Reactive protein at VAP onset, appropriate empirical treatment and ARDS.

Table S2. Significant univariate and multivariate Cox regression analyses of variables associated with 90-day mortality.

Variable	Univariate			Multivariable		
	HR	95% CI	p-value	HR	95% CI	p-value
ARDS	1.20	0.72–2.00	0.48	1.00	0.61–1.74	0.99
Age (+1)	1.02	1.00–1.03	0.014	1.02	1.01–1.04	0.003
Treatment with corticosteroids before VAP	1.86	1.09–3.18	0.023	1.87	1.09–3.21	0.024
Active neoplasm	1.86	1.13–3.05	0.014	1.89	1.13–3.17	0.015
Chronic heart disease	0.66	0.43–1.00	0.051	0.63	0.41–0.98	0.040
Chronic liver disease	2.07	1.32–3.24	0.001	1.73	1.09–2.75	0.021
SOFA at VAP onset (+1)	1.10	1.04–1.15	<0.001	1.06	1.00–1.13	0.041
Shock at VAP onset	2.21	1.49–3.28	<0.001	1.68	1.08–2.60	0.021

Significant univariate and multivariate Cox regression analyses of variables associated with 90-day mortality. Abbreviations: ARDS, acute respiratory distress syndrome; CI, confidence interval; HR, hazard ratio; SOFA, sequential organ failure assessment, VAP, ventilator-associated pneumonia. Data are shown as estimated HRs (95% CIs) of the explanatory variables in the 90-day mortality group. The HR is defined as the ratio of the hazard rates corresponding to the conditions described by two levels of an explanatory variable (the hazard rate is the risk of death [i.e., the probability of death], given that the patient has survived up to a specific time). The P-value is based on the null hypothesis that all HRs relating to an explanatory variable equal unity (no effect). [a] The variables analysed in the univariate analysis were age, sex, corticosteroid use before VAP, infection (respiratory and non-respiratory) before VAP, diabetes mellitus, chronic renal failure, chronic heart disease, chronic lung disease, active solid neoplasm, chronic liver disease, SOFA and shock at VAP onset, creatinine, hemoglobin, lymphocytes and C-Reactive protein at VAP onset, appropriate empirical treatment and ARDS.

Table S3. Sub-analysis of outcomes by timespans. Timespan 1 (2004–2007).

Outcomes	Patients with ventilator-associated pneumonia who developed acute respiratory distress syndrome n = 8	Patients with ventilator-associated pneumonia who did not develop acute respiratory distress syndrome n = 64	p-value
28-day mortality, n (%)	2 (25)	16 (25)	1
90-day mortality, n (%)	6 (75)	25 (40)	0.06
ICU mortality, n (%)	5 (62.5)	20 (31.3)	0.08
ICU length of stay (days), median (Q1; Q3)	38.5 (16; 54)	20 (14.5; 30)	0.13
Hospital length of stay (days), median (Q1; Q3)	45.5 (28.5; 58.5)	38.5 (21; 62)	0.72
28-day ventilator-free days, median (Q1; Q3)	0 (0; 16.5)	16 (0; 23)	0.15
Duration of mechanical ventilation (days), median (Q1; Q3)	40 (12; 60)	16 (19; 23)	0.045

Clinical outcomes of 72 patients with VAP who did and did not develop ARDS during 2004–2007. Abbreviations: ARDS, acute respiratory distress syndrome; ICU, intensive care unit; Q1, first quartile; Q3, third quartile; VAP, ventilator-associated pneumonia. Percentages calculated with non-missing data only.

Table S4. Sub-analysis of outcomes by timespans. Timespan 2 (2008–2010).

Outcomes	Patients with ventilator-associated pneumonia who developed acute respiratory distress syndrome n = 15	Patients with ventilator-associated pneumonia who did not develop acute respiratory distress syndrome n = 98	p-value
28-day mortality, n (%)	4 (26.7)	31 (31.6)	0.69
90-day mortality, n (%)	5 (33.3)	42 (43.8)	0.09
ICU mortality, n (%)	4 (26.7)	30 (30.6)	0.75
ICU Length of stay (days), median (Q1; Q3)	18 (12; 45)	16 (11; 27)	0.41
Hospital Length of stay (days), median (Q1; Q3)	33 (19; 123)	35.5 (17; 55)	0.52
28-day ventilator-free days, median (Q1; Q3)	0 (0; 20)	4 (0; 21)	0.52
Duration of mechanical ventilation (days), median (Q1; Q3)	18 (10; 39)	14 (9; 24)	0.50

Clinical outcomes of 113 patients with VAP who did and did not develop ARDS during 2008 - 2010. Abbreviations: ARDS, acute respiratory distress syndrome; ICU, intensive care unit; Q1, first quartile; Q3, third quartile; VAP, ventilator-associated pneumonia. Percentages calculated with non-missing data only.

Table S5. Sub-analysis of outcomes by timespans. Timespan 3 (2011–2013).

Outcomes	Patients with ventilator-associated pneumonia who developed acute respiratory distress syndrome n = 10	Patients with ventilator-associated pneumonia who did not develop acute respiratory distress syndrome n = 71	p-value
28-day mortality, n (%)	3 (30)	8 (11.3)	0.10
90-day mortality, n (%)	4 (44.4)	18 (26.1)	0.25
ICU mortality, n (%)	3 (30)	13 (18.3)	0.38
ICU Length of stay (days), median (Q1; Q3)	19.5 (12; 24)	17 (12; 31)	0.89
Hospital Length of stay (days), median (Q1; Q3)	41.5 (24; 91)	36 (24; 57)	0.58
28-day ventilator-free days, median (Q1; Q3)	10 (0; 19)	15 (0; 21)	0.40
Duration of mechanical ventilation (days), median (Q1; Q3)	11 (8; 18)	11 (8; 21)	0.77

Clinical outcomes of 81 patients with VAP who did and did not develop ARDS during 2011–2013. Abbreviations: ARDS, acute respiratory distress syndrome; ICU, intensive care unit; Q1, first quartile; Q3, third quartile; VAP, ventilator-associated pneumonia. Percentages calculated with non-missing data only.

Table S6. Sub-analysis of outcomes by timespans. Timespan 3 (2014–2016).

Outcomes	Patients with ventilator-associated pneumonia who developed acute respiratory distress syndrome n = 8	Patients with ventilator-associated pneumonia who did not develop acute respiratory distress syndrome n = 27	p-value
28-day mortality, n (%)	3 (37.5)	3 (11.1)	0.08
90-day mortality, n (%)	3 (37.5)	4 (14.8)	0.15
ICU mortality, n (%)	3 (37.5)	3 (11.1)	0.08
ICU Length of stay (days), median (Q1; Q3)	32.5 (17; 46.5)	28.5 (21; 49)	0.87
Hospital Length of stay (days), median (Q1; Q3)	53 (36; 55)	71 (32.5; 101)	0.66
28-day ventilator-free days, median (Q1; Q3)	7 (0; 18)	11 (0; 18)	0.89
Duration of mechanical ventilation (days), median (Q1; Q3)	23 (14; 29)	17 (13; 24)	0.40

Clinical outcomes of 35 patients with VAP who did and did not develop ARDS during 2014–2016. Abbreviations: ARDS, acute respiratory distress syndrome; ICU, intensive care unit; Q1, first quartile; Q3, third quartile; VAP, ventilator-associated pneumonia. Percentages calculated with non-missing data only.
